# EGFR-TKI耐药后的治疗选择

**DOI:** 10.3779/j.issn.1009-3419.2013.10.01

**Published:** 2013-10-20

**Authors:** 峻岭 李

**Affiliations:** 100021 北京，中国医学科学院北京协和医学院肿瘤医院内科 Department of Medical Oncology, Cancer Hospital (Institute), Chinese Academy of Medical Science, Peking Union Medical College, Beijing 100021, China

晚期肺腺癌的治疗已经进入了个体化治疗的时代，其标志是一些驱动基因的发现及相关药物的临床应用，产生了明显优于传统化疗的疗效，包括改善生活质量及延长患者的额外生存时间。主要包括表皮生长因子（epidermal growth factor receptor, EGFR）敏感突变的酪氨酸激酶抑制剂（tyrosine kinase inhibitor, TKI）治疗，以及间变性淋巴瘤激酶（anaplastic lymphoma kinase, ALK）阳性的克唑替尼治疗。TKI类药物治疗EGFR敏感突变的晚期肺腺癌可获得约70%的有效率及超过12个月的中位无进展生存期，该类药物口服给药，没有细胞毒类药物的血液学毒性，耐受性较好，患者的生活质量得到了提高。在使用TKI类药物治疗后，几乎所有的患者最终都会出现耐药而导致疾病进展，因此耐药的问题及耐药后的治疗选择成为临床医生主要关注与研究的内容。这组回顾性的数据来自中国医学科学院肿瘤医院，基本反映了临床肿瘤医生对于TKI类药耐药后肺癌治疗的具体临床实践。包括使用EGFR-TKI后出现耐药的进展模式、耐药后的化疗方案选择、孤立进展后的联合局部治疗以及多靶点药物的选择等。在此基础上，将要开展一些前瞻性的临床研究，以解决临床上遇到的问题。

